# Song Bu Li Decoction, a Traditional Uyghur Medicine, Protects Cell Death by Regulation of Oxidative Stress and Differentiation in Cultured PC12 Cells

**DOI:** 10.1155/2013/687958

**Published:** 2013-09-28

**Authors:** Maitinuer Maiwulanjiang, Kevin Y. Zhu, Jianping Chen, Abudureyimu Miernisha, Sherry L. Xu, Crystal Y. Q. Du, Kitty K. M. Lau, Roy C. Y. Choi, Tina T. X. Dong, Haji A. Aisa, Karl W. K. Tsim

**Affiliations:** ^1^Division of Life Science and Centre for Chinese Medicine, The Hong Kong University of Science and Technology, Clear Water Bay Road, Hong Kong; ^2^Xinjiang Key Laboratory of Plant Resources and Natural Products Chemistry, Xinjiang Technical Institute of Physics and Chemistry, Chinese Academy of Sciences, Urumqi 830011, China

## Abstract

Song Bu Li decoction (SBL) is a traditional Uyghur medicinal herbal preparation, containing Nardostachyos Radix et Rhizoma. Recently, SBL is being used to treat neurological disorders (insomnia and neurasthenia) and heart disorders (arrhythmia and palpitation). Although this herbal extract has been used for many years, there is no scientific basis about its effectiveness. Here, we aimed to evaluate the protective and differentiating activities of SBL in cultured PC12 cells. The pretreatment of SBL protected the cell against tBHP-induced cell death in a dose-dependent manner. In parallel, SBL suppressed intracellular reactive oxygen species (ROS) formation. The transcriptional activity of antioxidant response element (ARE), as well as the key antioxidative stress proteins, was induced in dose-dependent manner by SBL in the cultures. In cultured PC12 cells, the expression of neurofilament, a protein marker for neuronal differentiation, was markedly induced by applied herbal extract. Moreover, the nerve growth factor- (NGF-) induced neurite outgrowth in cultured PC12 cells was significantly potentiated by the cotreatment of SBL. In accord, the expression of neurofilament was increased in the treatment of SBL. These results therefore suggested a possible role of SBL by its effect on neuron differentiation and protection against oxidative stress.

## 1. Introduction 

Traditional Uyghur medicine (TUM), one of the main medicinal systems in central Asia, is based on four humors: fire, air, water, and earth, which generates four different body fluids: blood, phlegm, yellow bile, and black bile [1]. The main ingredients of TUM are flowers, seeds, fruits, minerals, and animal compartments. According to the TUM theory, diseases or impairments are resulted from imbalance between the four body fluids. TUM herbal formulation could regulate the balance of body fluids and cure diseases [2]. Song Bu Li decoction (SBL), a TUM formula described in Sherhi Alkanun by Emam Durdin during AD 840-1212, consists of only one herb named Nardostachyos Radix et Rhizoma (NRR, a root and rhizome of *Nardostachys jatamansi*). Recently, SBL has been used to treat neurological disorders (insomnia and neurasthenia) and heart disorders (palpitation and arrhythmia) in Xinjiang of China. According to ancient method of preparations of TUM [2], two methods of SBL preparation are commonly used in Xinjiang: (i) NRR is boiled with water, and the vapor generated is being collected as the volatile components of NRR. Condensed vapor and extracted water are mixed and (ii) NRR is boiled in water and filtered, and only the water extract is being used for treatment. Both preparation methods of SBL generate a problem of quality control and efficacy. 

Neurasthenia is defined as a condition with symptoms of fatigue, forgetfulness, sleepless, anxiety, and depressed mood [3, 4], and the pathogenesis of neurasthenia still remains unknown [5]. During the brain development, neuronal stem cells undergo a stage called neurogenesis in which immature neurons grow, differentiate, and survive. However, for patients with neurasthenia and depression, the normal neurogenesis would be impaired, that is, the inability for neurons to differentiate normally. Amongst different causes of neuronal cell death, reactive oxygen species (ROS) mediated oxidative stress is one of the major origins of many neurological disorders [6–8]. The excess generation of ROS damages cells by peroxidizing lipids and disrupting structural proteins, enzymes, and nucleic acids [9, 10]. In defending the stress, antioxidant response element (ARE), located upstream of various genes, could regulate the expression of antioxidative stress proteins [11–13]. 

Neuronal differentiation of PC12 cells, mediated by nerve growth factor (NGF), shows the morphological change of cells possessing neurites. In addition, the neuronal differentiation could be determined biochemically by analyzing the expression of neurofilaments (NFs) that are the major structural components of differentiated neurons [14, 15]. Three mammalian neurofilament subunits, NF68, NF160, and NF200 are believed to form heterodimers in making the structural domain of neurites [16]. The differentiation status of neuron is also a critical parameter of neuron survival. 

Having the questions of SBL efficacy under two distinct preparative methods, we aimed to establish quality control parameters of the herbal preparation and to reveal the role of SBL in preventing neuronal cell death. Chemical fingerprint and quantitation of ingredients, including ferulic acid, linarin, and volatile oil, were developed for quality control. In the bioassay, the role of SBL in preventing *tert*-Butyl hydroperoxide- (tBHP-) induced cell death as well as the gene activation of antioxidative stress proteins was determined. Lastly, the length of neurites and the expression of neurofilaments were determined in PC12 cells under the treatment of SBL. 

## 2. Materials and Methods

### 2.1. Plant Materials and Preparation of SBL

NRR, the root and rhizome of *N. jatamansi*, was purchased from Hong Kong herbal market (Wong Chak Kee Co.). The authentication of plant material was performed by Dr. Tina T. X. Dong according to their morphological characteristics. The voucher specimens were deposited in the Centre for Chinese Medicine R&D at The Hong Kong University of Science and Technology. The herb NRR was minced and soaked in water in the proportion of 1 : 10 (w/v) overnight. The mixture was submitted to hydrodistillation in a Clevenger-type apparatus for 4 hours. Water extract and volatile oil of NRR were obtained at the same time. Volatile oil was dried over anhydrous sodium and stored at −20°C. The resulting water extract was filtered, vacuum-dried to powder, and kept at −20°C. This extract was considered as an NRR extract. The extraction efficiency reached over 95% within 4 hours. For SBL preparation, 1 g of dried NRR powder was dissolved in 20 mL DMSO and sonicated for 30 min. The extract was centrifuged at 13,200 rpm at 4°C for 5 min, and 170 *μ*L of volatile oil was added onto the supernatant as the final SBL decoction. 

### 2.2. Chemicals and Reagents

Ferulic acid (>98%), *tert*-Butyl hydroperoxide (tBHP) (>98%), *tert*-Butylhydroquinone (tBHQ) (>98%), 3-(4,5-dimethylthiazol-2-yl)-2,5-diphenyl tetrazolium bromide (MTT) (>98%) were purchased from Sigma Chemical Co. (St. Louis, MO, USA). Linarin (>98%) was kindly provided by Testing Laboratory for Chinese Medicine (Hong Kong, China). HPLC-grade acetonitrile and methanol were purchased from Merck (Darmstadt, Germany). Ultra-pure water was prepared from a Milli-Q purification system (Millipore, Molsheim, France).

### 2.3. GC-MS Analysis

Agilent 7000 GC-MS series system (Waldbronn, Germany) equipped with an Agilent 7890A gas chromatography and GC-QQQ Mass Hunter workstation software was adopted. The extract was separated in an Agilent HP-5MS capillary column (250 *μ*m × 30 m × 0.25 *μ*m) with controlled temperature at 100°C in the initial stage, and the temperature was adjusted to 280°C at the rate of 5°C/min. Pulsed splitless injection was conducted by injecting 1 *μ*L of the sample extract. Helium was used as carrier gas at a flow rate of 2.25 mL/min; nitrogen was used as the collision gas at a flow rate of 1.5 mL/min. The spectrometer was operated in a full-scan electron-impact (EI) mode, and the ionization energy was 70 eV. The inlet and ionization source temperatures were 250°C and 230°C, respectively. The solvent delay time was 3.5 min. Retention indices of all compounds were determined according to the Kovats method using *n*-alkanes (Sigma) as standards. Identification of the volatile compounds was confirmed by comparing the mass spectra with the Kovats retention indices.

### 2.4. HPLC-DAD Analysis

HPLC-DAD analysis was conducted with an Agilent HPLC 1200 series system (Agilent Waldbronn, Germany), which was equipped with a degasser, a binary pump, an autosampler, a diode array detector (DAD), and thermo-stated column compartment. Chromatographic separation was carried out on an Intersil C18 column (particle size 5 *μ*m, 4.6 × 250 mm) with water (as solvent A) and acetonitrile (as solvent B) as the mobile phase at flow rate of 1.0 mL/min at room temperature using the following gradient program: 0–20 min, isocratic gradient 19-19% (B); 20–25 min, linear gradient 19–25% (B); 25–45 min, linear gradient 25–35% (B); and 45–55 min, linear gradient 35–55% (B). A preequilibration period of 10 min was used between each run. The injection volume was 10 *μ*L. The UV detector wavelength was set to 334 nm with full spectral scanning from 190 to 400 nm. 

### 2.5. PC12 Cell Culture

Pheochromocytoma PC12 cells, a cell line derived from rat adrenal medulla, were obtained from American Type Culture Collection (ATCC, Manassas, VA) and maintained in Dulbecco's modified Eagle's medium (DMEM) supplemented with 6% fetal bovine serum, 6% horse serum, 100 units/mL penicillin, and 100 *μ*g/mL of streptomycin in a humidified CO_2_ (7.5%) incubator at 37°C. Culture reagents were from Invitrogen (Carlsbad, CA). For the differentiation assay, cultured PC12 cells were serum starved for 4 hours in DMEM supplemented with 1% fetal bovine serum, 1% horse serum, and penicillin-streptomycin, and then they were treated with the SBL and/or other reagents for 72 hours. 

### 2.6. MTT and ROS Formation Assay

PC12 cell viability was assayed by reduction of MTT [3-(4,5-dimethylthiazol-2-yl)-2,5-diphenyl tetrazolium bromide] reagent. Cells (2 × 10^4^ cells/well) were plated in 96-well plate and pretreated with different concentrations of SBL and/or other reagents for 24 hours. Then, the cells were treated with 150 *μ*M tBHP for 3 hours. The cultures were then treated with MTT solution for 1 hour, and the optical density was measured using spectrophotometer at 570 nm. The determination of ROS level in cell cultures was performed according to Zhu et al. [17]. In brief, cultured PC12 cells in a 96-well plate were pretreated with different concentrations of SBL and/or other reagents for 24 hours and labeled by 100 *μ*M DCFH-DA (Sigma) in HBSS for 1 hour at room temperature. Cultures were then treated with 100 *μ*M tBHP for 1 hour. The amount of intracellular tBHP-induced ROS formation was detected by fluorometric measurement with excitation at 485 nm and emission at 530 nm (Spectra max Gemini XS, Molecular Devices Crop., Sunnyvale, CA).

### 2.7. DNA Construction and Transfection

The pGL4.37[*luc2P*/ARE/Hygro] vector contains four copies of an antioxidant response element (ARE; 5′-TGACnnnGCA-3′) that drives transcription of the luciferase reporter gene *luc2P* (*Photinus pyralis*)*. Luc 2P* is a synthetically-derived luciferase sequence with humanized codon optimization that is designed for high expression and reduced anomalous transcription. The *luc2P* gene contains hPEST, a protein destabilization sequence, which allows *luc2P* protein levels to respond more quickly than those of *luc2* to induce transcription. Cultured PC12 cells were transfected with pARE-Luc (Promega, Fitchburg, WI) by Lipofectamine 2000 (Invitrogen) according to the manufacturer's instructions. The transfection efficiency was ~60%, as determined by another control plasmid having *β*-galactosidase, under a cytomegalovirus enhancer promoter.

### 2.8. Luciferase Activity

PC12 cells, cultured in 24-well plate (1 × 10^5^ cells/well), were treated with SBL and/or other reagents for 24 hours. Afterward, the medium was aspirated, and cultures were washed by ice-cold PBS. The cells were lysed by a buffer containing 0.2% Triton X-100, 1 mM dithiothreitol, and 100 mM potassium phosphate buffer (pH 7.8) at 4°C. Followed by centrifugation at 13,200 rpm for 10 min at 4°C, the supernatant was collected and used to perform luciferase assay (Tropix Inc., Bedford, MA); the activity was normalized by amount of protein and the activity of *β*-galactosidase (a control plasmid).

### 2.9. Polymerase Chain Reaction (PCR) Analysis

PC12 cells were treated with SBL and/or other reagents for 24 hours. Total RNAs were isolated by TRIzol reagent (Invitrogen) and reverse-transcribed by Moloney Murine Leukemia Virus Reverse Transcriptase (Invitrogen) according to the manufacturer's instruction. Real-time PCR was performed by using SYBR green master mix and ROX reference dye according to the manufacturer's instruction (Applied Bioscience, Foster City, CA). The primers were as follows: 5′-GAC CTT GCT TTC CAT CAC CAC CGG-3′ and 5′-GTA GAG TGG TGA CTC CTC CCA GAC-3′ for NAD(P)H quinone oxidoreductase (NQO1; 241 bp); 5′-CCT GCT GTG TGA TGC CAC CAG ATT TT-3′ and 5′-TCT GCT TTT CAC GAT GAC CGA GTA CC-3′ for glutamate-cysteine ligase modulatory subunit (GCLM; 197 bp); 5′-CGT GGA CAC CCG ATG CAG TAT TCT G-3′ and 5′-GGG TCG CTT TTA CCT CCA CTG TAC T-3′ for glutamate-cysteine catalytic subunit (GCLC; 261 bp); 5′-CCT GGG CAT CTG AAA CCT TTT GAG AC-3′ and 5′-GCG AGC CAC ATA GGC AGA GAG C-3′ for glutathione *S*-transferase (GST; 180 bp). Glyceraldehyde 3-phosphate dehydrogenase (GAPDH) was used as an internal control in all cases, and its primer sequence was 5′-AAC GGA TTT GGC CGT ATT GG-3′ and 5′-CTT CCC GTT CAG CTC TGG G-3′ (657 bp). SYBR green signal was detected by Mx3000ptm multiplex quantitative PCR machine (Applied Bioscience, Foster City, CA). The transcript levels were quantified by using ΔΔ*C*_*t*_ value method [18]. Calculations were done using the *C*_*t*_ value of GAPDH to normalize the *C*_*t*_ value of target genes in each sample to obtain the Δ*C*_*t*_ values which were used to compare among different samples. PCR products were analyzed by gel electrophoresis and melting curve analysis to confirm specific amplifications. 

### 2.10. Neurite Outgrowth Assay

PC12 cells were treated with SBL or NGF for 72 hours. A light microscope (Diagnostic Instruments, Sterling Heights, MI) equipped with a phase-contrast condenser (Zeiss), 10X objective lens, and a digital camera (Diagnostic Instruments) was used to capture the image with manual setting. For analyzing the number and length of neurite, approximately 100 cells were counted from at least 10 randomly chosen visual fields for each culture. Using the Photoshop software, the number and length of neurite were analyzed. The cells were scored as differentiated if one or more neurites were longer than the diameter of the cell body, and they were also classified into different groups according to the lengths of neurites, which are <15 *μ*m, 15–30 *μ*m, and >30 *μ*m.

### 2.11. Western Blot Analysis

After the indicated time of treatment, cultures were collected in the high salt lysis buffer (10 mM HEPES, pH 7.5, 1 M NaCl, 1 mM EDTA, 1 mM EGTA, 0.5% Triton X-100, 5 mM benzamidine HCl, 10 *μ*M aprotinin, 10 *μ*M leupeptin) and were analyzed immediately or stored frozen at −20°C. Proteins were separated on the 8% SDS-polyacrylamide gels and transferred to the nitrocellulose membrane. Successful transfer and equal loading of samples were confirmed by staining Ponceau-*S*. The nitrocellulose membrane was blocked with 5% fat-free milk in TBS-T (20 mM Tris base, 137 mM NaCl, 0.1% Tween-20, pH 7.6) for 2 hours at room temperature, and then it was incubated in the primary antibody diluted in 2.5% fat-free milk in TBS-T for 16 hours at 4°C. The primary antibodies used were antiNF200 (Sigma), antiNF160 (Sigma), antiNF68 (Sigma), and antiGAPDH (Calbiochem, Germany). After that, the nitrocellulose was rinsed with TBS-T and incubated for 2 hours at the room temperature in horseradish peroxidase conjugated goat antimouse secondary antibody (Invitrogen) diluted in the 2.5% fat-free milk in TBS-T. After intensive washing with TBS-T, the immune complexes were visualized using the enhanced chemiluminescence (ECL) method (GE Healthcare, Piscataway, NJ). The intensities of the bands in the control and different samples were run on the same gel and under strictly standardized ECL conditions and compared on an image analyzer using a calibration plot constructed from a parallel gel with serial dilutions of one of the samples.

### 2.12. Other Assays

The protein concentrations were measured routinely by Bradford's method with kit from Bio-Rad Laboratories (Hercules, CA). Statistical tests were done by using one-way analysis by student's *t*-test on Prism 4.00. Differences from basal or control values (as shown in the plots) were classed as significant (*) where *P* < 0.05, (**) where *P* < 0.01.

## 3. Results

### 3.1. Chemical Analysis of SBL

**Figure 1:**
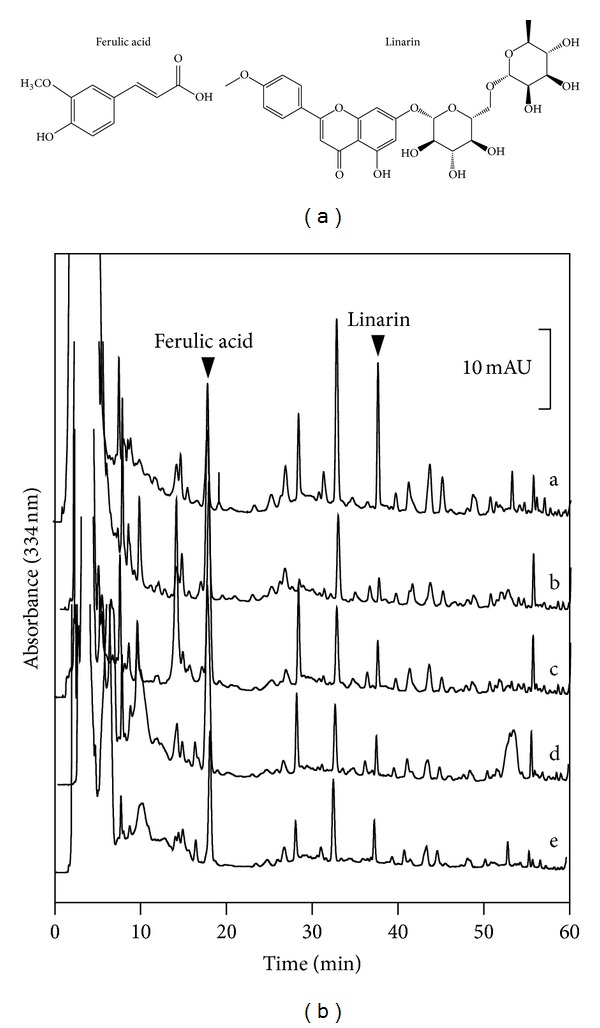
Chemical standardization of SBL by HPLC fingerprint analysis. Chemical structures of ferulic acid and linarin were shown (a). In the HPLC chromatogram at an absorbance of 334 nm (b), the peaks corresponding to ferulic acid and linarin in SBL are indicated by arrowheads. The fingerprints of different batches of NRR extracts (a–e) that were collected from (a) Songpan, (b) Abei, (c) Hongyuan, (d) Wing Lee Hong (HK), and (e) Wong Chak Kee (HK) of China are shown. The chromatographic method was described in Section 2. Representative chromatograms are shown, *n* = 3.

According to the ancient method of preparation, two extracts were obtained from NRR extraction: (i) NRR extract without volatile oil, that is, NRR extract; and (ii) NRR plus volatile oil, that is, SBL. Both NRR and oil extracts were chemically standardized. The volatile oil from NRR was analyzed by GC-MS: 14 components were identified, which accounted for 79.1% of the total volatile oil. The amount of volatile oil within NRR was 2.1 ± 0.28% (*n* = 4), and the relative amounts of each chemical were given (Table 1). The major components of the oil were calarene (37.9%), *β*-maaliene (7.6%), and 9-aristolene (5.1%). Thus, a standardized NRR volatile oil should contain at least the chemicals as stated here. To standardize the water extract of NRR, HPLC fingerprint was generated. The amount of NRR extract from crude herb was 13.34 ± 0.45% (*n* = 4). The fingerprints of five different batches of NRR extract were compared, and this showed the consistence of the herbal extract (Figure 1). Ferulic acid and linarin were determined as chemical markers: these chemicals were reported to have known biological functions as described previously [19–21]. The quantification of chemical markers was carried out by measuring the peak area according to the regression equation (see Table 1 in Supplementary Material available online at http://dx.doi.org/10.1155/2013/687958). Using the established HPLC method, the calibration curves of ferulic acid and linarin exhibited good linearity within a specific range of concentration. The correlation coefficients (*r* ^2^) of those chemical markers were higher than 0.999. The limit of detection (LOD) and limit of quantification (LOQ) were determined at S/N of 3 and 10, respectively (Supplementary Table 1). The precision and repeatability of the chemical measurement were excellent, having a relative standard deviation (RSD) <5% (Supplementary Table 2). The recovery experiment was carried out to evaluate the method accuracy. The recoveries of ferulic acid and linarin were 99.31% and 100.12%, respectively. Thus, the employed HPLC method was validated by in performing the quantitative analysis. Here, we recommended that a standardized NRR extract should contain at least 24.8 *μ*g ferulic acid and 114.9 *μ*g linarin in 1 g of dried NRR extract. A well-standardized SBL was prepared with the mixture of NRR extract and volatile oil in a ratio of 6 (NRR water extract; w) : 1 (NRR volatile oil; v).

### 3.2. SBL on tBHP-Treated PC12 Cells

**Figure 2:**
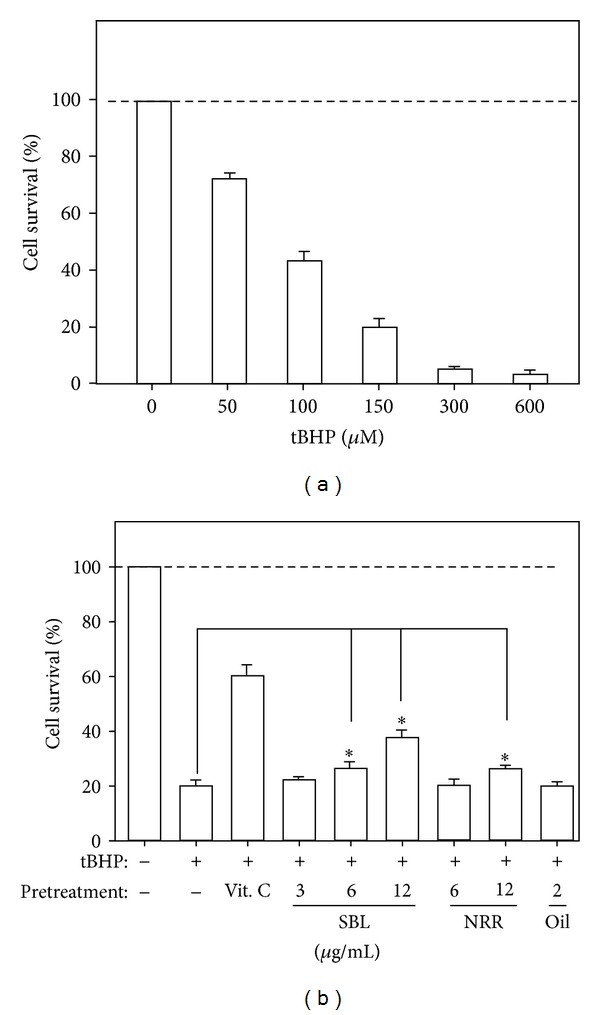
SBL prevents cell death in tBHP-treated PC12 cells. (a) Cultured PC12 cells were treated with tBHP (0–600 *μ*M) for 3 hours to determine the cytotoxicity of tBHP by cell viability assay. (b) PC12 cells were pretreated with SBL (3–12 *μ*g/mL), NRR (6 and 12 *μ*g/mL) and volatile oil (2 *μ*g/mL) for 24 hours before the addition of tBHP (150 *μ*M) for 3 hours. The neuroprotective effect of SBL by cell viability assay was shown. Vitamin C (1 mM) served as a positive control. The viability of PC12 cells is shown in percentage of MTT value relative to normal control. Data are expressed as Mean ± SEM, where *n* = 5, each with triplicate samples. *P < 0.05 and **P < 0.01 as compared to the group (with tBHP alone).

To elucidate the function of NRR extract and SBL, the herbal extract was applied onto cultured PC12 cells. Firstly, the concentrations (3–12 *μ*g/mL) of applied SBL did not show cytotoxicity or proliferating effect on the cultures (Supplementary Figure 1). The cell viability, determined by MTT assay, was significantly decreased by tBHP in dose-dependent manner (Figure 2(a)). Cultured PC12 cells were pretreated with SBL (3–12 *μ*g/mL) for 24 hours before the challenged tBHP. Higher concentrations (6 and 12 *μ*g/mL) of SBL showed significant protection effect against the tBHP-induced cell death (Figure 2(b)). The applications of NRR extract (6 *μ*g/mL) and volatile oil (2 *μ*g/mL) in the cultures did not show any effect. Higher dose of NRR extract (12 *μ*g/mL) showed the protection effect which, however, was lower than that of SBL (Figure 2(b)). Thus, the protection effect of SBL was better than that of NRR extract and volatile oil. The tBHP-induced cell mortality in PC12 cells was markedly reduced by the pretreatment of vitamin C, a positive control (Figure 2(b)). 

**Figure 3:**
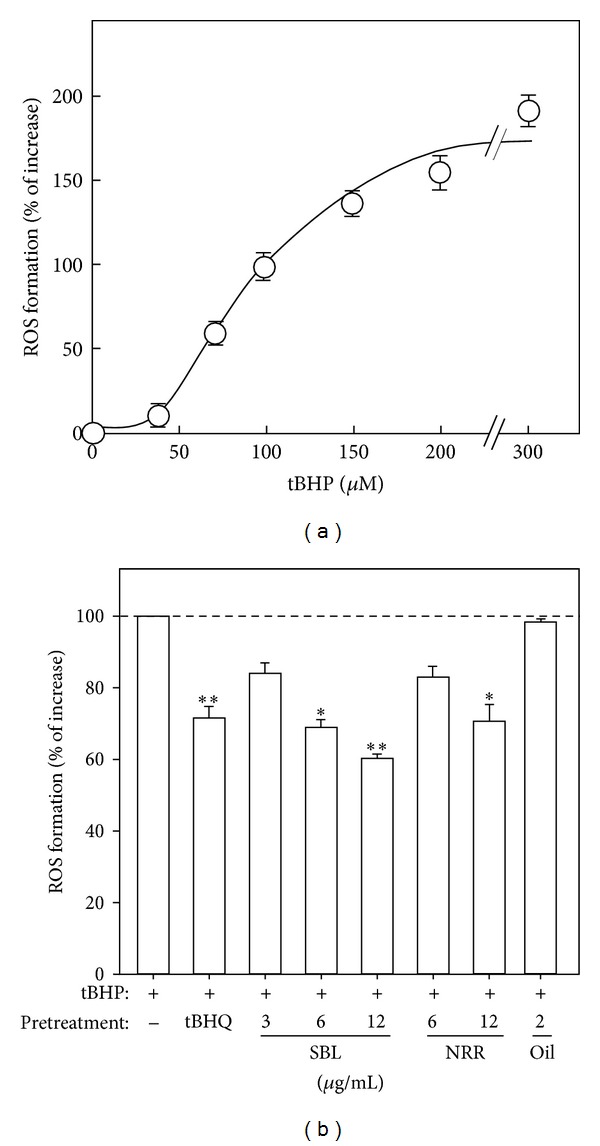
SBL suppresses the tBHP-induced ROS formation in PC12 cells. (a) Cultured PC12 cells were exposed to tBHP (0–300 *μ*M) for 1 hour. The level of intracellular ROS formation was measured by fluorescence method. The results are shown in percentage of increase in ROS formation relative to the control (without tBHP). (b) Cultured PC12 cells were pretreated with SBL (3–12 *μ*g/mL), NRR (6 and 12 *μ*g/mL), and volatile oil (2 *μ*g/mL) for 24 hours and then exposed to tBHP (100 *μ*M) for 1 hour. The pretreatment of tBHQ (3 *μ*M) was used for comparison. The results are shown in percentage of ROS formation relative to the control (with tBHP alone). Data are expressed as Mean ± SEM, where *n* = 5, each with triplicate samples. *P < 0.05 and **P < 0.01 as compared to the group (with tBHP alone).

The formation of ROS is one of the crucial causes in inducing neuronal cell death. By determining the formation of ROS in tBHP-treated PC12 cells, the role of SBL was analyzed. Application of tBHP in cultured PC12 cells induced the ROS formation in dose-dependent manner (Figure 3(a)). The tBHP-induced ROS formation was reduced by ~25% after the pretreatment of tBHQ, a known antioxidant. SBL, NRR extract and volatile oil were applied for 24 hours before the addition of tBHP (100 *μ*M), and then the cultures were subjected to the determination of intracellular ROS formation. Pretreatment of SBL reduced tBHP-induced ROS formation in dose-dependent manner: the maximal reduction at ~40% was revealed after the treatment of 12 *μ*g/mL SBL (Figure 3(b)). Although NRR extract (12 and 6 *μ*g/mL) showed the reduction effect in ROS formation, compared with SBL, the reduction efficacy was not as good as SBL. Pretreatment of volatile oil (2 *μ*g/mL) did not show any reduction effect. 

**Figure 4:**
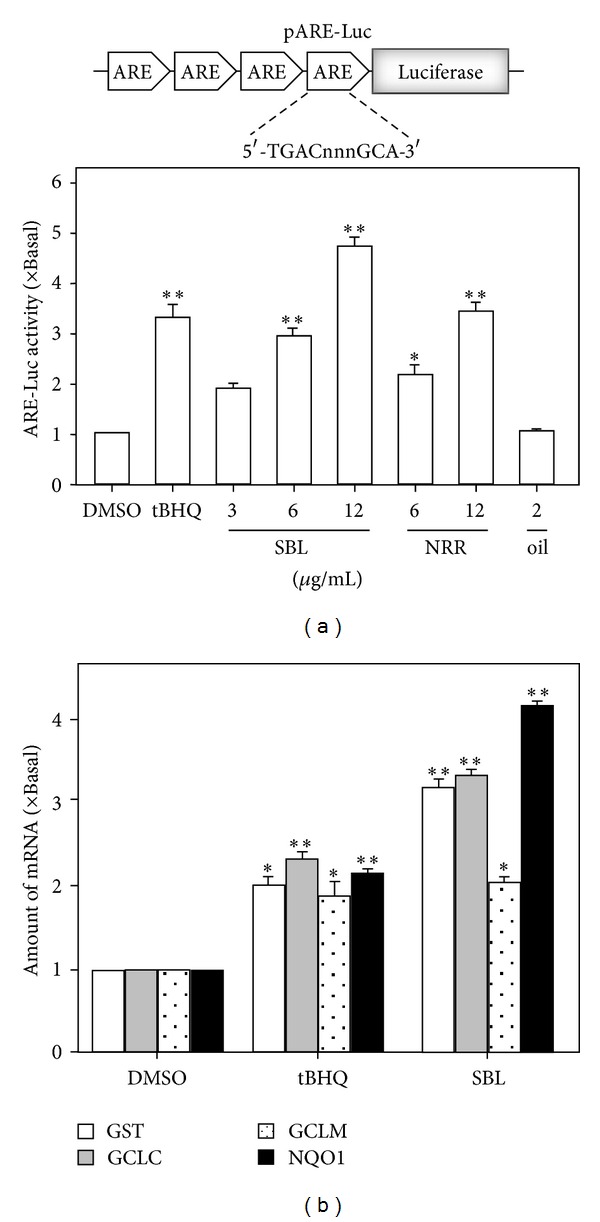
SBL induces ARE transcriptional activity and detoxifying enzymes in PC12 cells. (a) Four repeats of antioxidant responsive element (ARE: 5′-TGACnnnGCA-3′) tagged with luciferase-reporter vector called pARE-luc (upper panel). This reporter was stably transfected to PC12 cells, which were treated with SBL, NRR, and volatile oil for 24 hours (lower panel). tBHQ (3 *μ*M) was used as a positive control. (b) Cultured PC12 cells treated with SBL (12 *μ*g/mL) and tBHQ (3 *μ*M) for 24 hours. Total RNAs were isolated from cultured PC12 cells and then reversed transcribed into cDNAs for the detection of mRNAs encoding for GST, GCLC, GCLM, and NQO1 by real-time PCR analysis. The GAPDH served as internal control. Values are expressed as the fold of increase to basal reading (untreated culture), and in Mean ± SEM, where *n* = 4, each with triplicate samples. *P < 0.05 and **P < 0.01.

ARE is a *cis*-acting regulatory element or enhancer sequence, which is found in promoter regions of genes encoding detoxifying enzymes and antioxidant proteins [12]. To study the signaling mechanism of SBL in neuroprotection, the transcriptional activity of ARE, triggered by SBL, was studied. PC12 cells were stably transfected with a promoter-reporter construct containing four repeats of ARE tagged with luciferace reporter gene (pARE-Luc) (Figure 4(a)). The pARE-Luc stably transfected PC12 cells were treated with SBL, NRR extract, and volatile oil for 24 hours, and then the cell lysates were collected to determine the luciferase activity. The application of SBL increased the transcriptional activity of pARE-Luc in dose-dependent manner, in which the maximal induction at ~5-fold was revealed at 12 *μ*g/mL SBL (Figure 4(a)). NRR extract showed similar gene activation but at lower extend. NRR volatile oil could not increase the luciferase activity (Figure 4(a)). Based on the results of pARE-Luc activation, we investigated the role of SBL in the expression of detoxifying enzymes that were stimulated by the responsive element ARE. The ARE-derived genes, including GST, GCLC, GCLM, and NQO1, were investigated via real-time qPCR. Cultured PC12 cells were treated with SBL (12 *μ*g/mL) for 24 hours. The levels of GST and GCLC mRNA were increased by ~3-fold with treatment of SBL. The expression level of GCLC was increased by ~2-fold. In the regulation of NQO1 expression, SBL showed a robust induction of NQO1 mRNA by over ~4-fold (Figure 4(b)). This gene induction was better than that of tBHQ (3 *μ*M), a positive control.

### 3.3. SBL on the Differentiation of PC12 Cells

**Figure 5:**
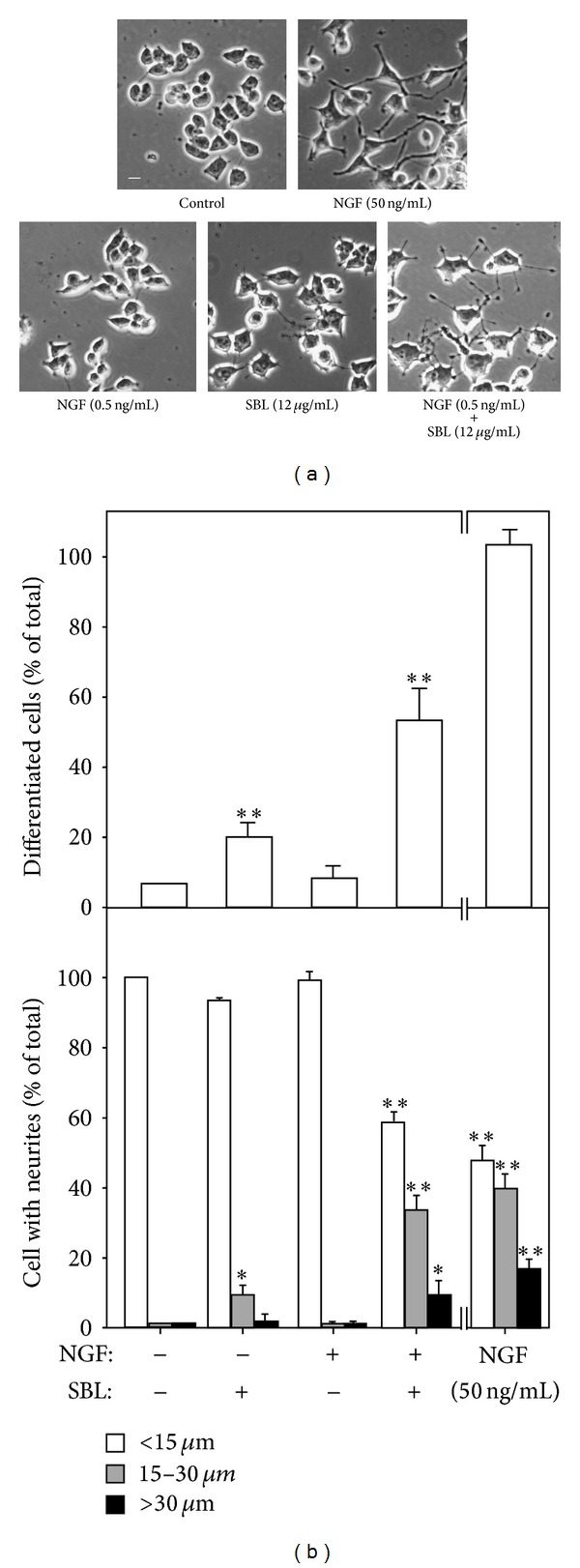
SBL potentiates the NGF-induced neurite outgrowth. (a) PC12 cells were treated with NGF (0.5 ng/mL and 50 ng/mL), SBL (12 *μ*g/mL), and NGF (0.5 ng/mL) + SBL (12 *μ*g/mL) for 72 hours. The cultures were fixed, and extension of neurite outgrowth was revealed. Bar = 10 *μ*m. (b) The % of differentiated cell (upper panel) and length of neurite (lower panel) were counted as described in Section 2. Values are expressed as % of cells in 100 counted cells. Mean ± SEM, where *n* = 4. *P < 0.05 and **P < 0.01 compared with control.

Neuronal differentiation effect of SBL on cultured PC12 cells was analyzed. After the treatment of NGF (50 ng/mL), the morphological change was observed. Longer neurites were protruded from the cell bodies (Figure 5(a)). This NGF treatment resulted in a 100% conversion of differentiated cells containing significant extension of neruites (Figure 5(b)). To evaluate the efficacies of SBL on PC12 differentiation, cells were treated with SBL (12 *μ*g/mL) for 72 hours to induce a slight increase of neurite outgrowth with a conversion of differentiated cell by ~20% (Figures 5(a) and 5(b)). Here, we aimed to determine the treatment of SBL together with low dose of NGF. A low concentration of NGF at 0.5 ng/mL failed to induce the neurite extension (Figure 5(a)). However, the cotreatment of SBL with low dose of NGF significantly potentiated the number of differentiated cell (at ~60%) as well as the neurite outgrowth (Figure 5(b)). 

**Figure 6:**
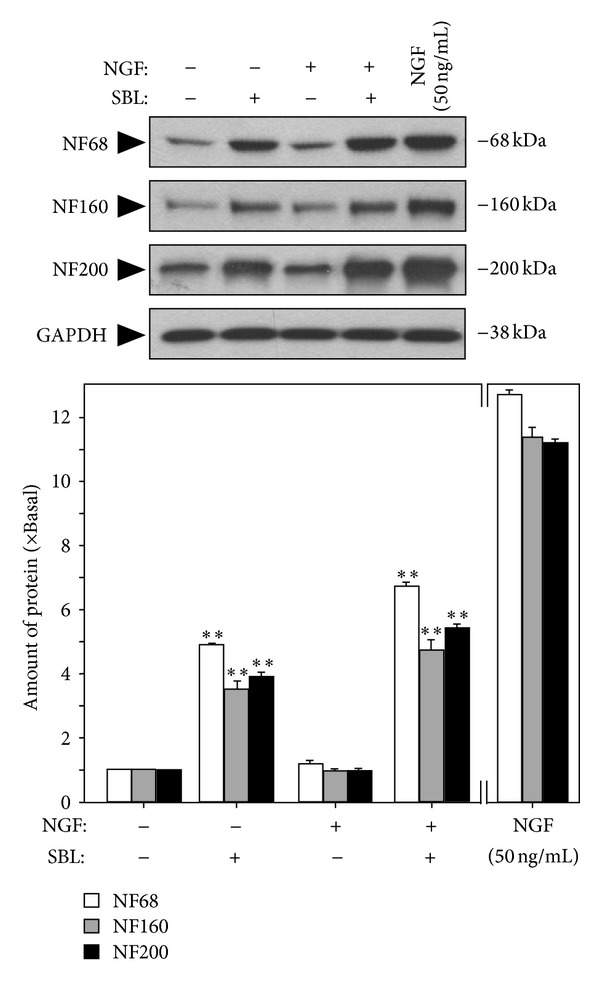
SBL potentiates the expression of neurofilaments. Cultured PC12 cells were treated with control, NGF (0.5 ng/mL or 50 ng/mL), SBL (12 *μ*g/mL), and NGF (0.5 ng/mL) + SBL (12 *μ*g/mL) for 72 hours. The cultures were collected to determine the change of neurofilaments expression (NF68, NF160, and NF200). NGF at 50 ng/mL was set as the positive control. GAPDH served as a loading control. Representative images are shown (upper panel). The lower panel shows the quantification from the blots by a densitometer. Values are expressed as the fold of change (x Basal) against the control (no treatment; set as 1), and in Mean ± SEM, where *n* = 4.

The SBL-induced neurofilament expression was also determined. After the treatment of SBL for 72 hours, the cells were collected to perform western blot analysis to determine the expressions of NF68, NF160, and NF200. By treatment of 12 *μ*g/mL SBL, the expression of NF68 was increased by ~5-fold, while NF160 and NF200 were altered by ~4-fold (Figure 6). The induction of neurofilament was also revealed in the cotreatment of SBL with low dose of NGF. High dose of NGF, a positive control, showed robust protein induction. The expression of control protein GAPDH remained unchanged (Figure 6). 

## 4. Discussion 

SBL decoction is one of the simplest herbal medicinal preparations of TUM containing only one herb NRR. Based on TUM theory, SBL can be used to treat functional reduction of brain and heart caused by wet-cold and phlegm [2, 22]. In line to ancient usage of SBL, here, we provided different lines of evidence to support the role of SBL in brain functions. First, SBL prevented the tBHP-induced cell death, which most likely was mediated by a reduction of ROS formation. Second, SBL induced the pARE-Luc activity as well as the gene activation of the key antioxidative stress proteins. Third, SBL induced differentiation of PC12 and the extension of neurites. More robustly, the cotreatment of SBL with NGF significantly induced the neuronal differentiation of PC12 cells. In parallel, the SBL-induced PC12 differentiation was shown to have a marked increase of neurofilament expression. These results strongly support the notion of SBL in treating neurasthenia clinically. 

According to TUM practitioner's practice, two SBL preparation methods are performed in Xinjiang. The only difference between these two preparation methods is the collection of the vapor (i.e., volatile oil) during the boiling process. Indeed, NRR is well known to possess high amount of volatile oil [23]. To guarantee the consistency of SBL preparation chemically, we standardized both water extract and volatile oil of NRR. The amount of NRR extract and volatile oil within NRR by weight were 13.34% and 2.1%, respectively. According to this ratio, NRR water extract and volatile oil were mixed together in the ratio of 6 : 1 (w : v) to generate an authentic decoction of SBL. Here, we compared the biological efficacy of NRR extract, volatile oil, and SBL. Our results suggested that SBL in general showed better effects than that of NRR extract. Interestingly, volatile oil alone had no biological effect. This synergetic effect of volatile oil and NRR water extract was fully revealed in the case of SBL. Thus, a completed preparation (i.e., NRR water extract + volatile oil) of SBL is recommended for decoction preparation. 

The redox-sensitive transcription factor NF-E2-related factor-2 (Nrf2) has been demonstrated to be a critical transcription factor that binds to antioxidant response element (ARE) in the promoter region of genes that code for phase II detoxifying enzymes in several types of cells. Activation of phase II detoxifying enzymes, such as GST, GCLM, GCLC, and NQO1, by phytochemicals resulted in detoxifying ROS [24]. As demonstrated here, the antioxidative role of SBL could be in several aspects: (i) suppressing the formation of ROS, (ii) inducing the gene activation of pARE-Luc, and (iii) inducing antioxidative stress proteins. Amongst different antioxidative stress proteins, GST is involved in the detoxification of free radicals through catalyzing the conjugation of GSH: this is considered as one of the key enzymes associated with chemoprevention [11]. GSH is synthesized by the consecutive action of two enzymes, GCLC and GCLM [12]. Moreover, NQO1, a well-known Nrf2-ARE regulated enzyme, protects cells against deleterious reactive semiquinones by converting exogenous quinones into hydroquinones through two-electron reduction pathway [25]. Thus, the pretreatment of SBL protected the cells against oxidative-stress-induced cell death by scavenging ROS and activating the Nrf2-ARE self-defense mechanism. 

Differentiation is a vital process for maturation of neuronal cells. When neurons differentiate, the neuron cell body (soma) would protrude long neurites in order to connect with other neurons to form synapses. Most of the neurological disorders are caused by the deficit of synaptic formation. During the neuronal differentiation, the expression level of neurofilaments and the neuronal cell specific cytoskeleton proteins, including 68, 160, 200 (kDa), were increased [14, 15]. In general, NF68 is expressed at the beginning of neurite outgrowth; then, NF160 is expressed shortly after with the emergence of neurite formation, and NF200 is expressed later when axonal radial growth is required for nervous system maturation. After the treatment of SBL, both neuron differentiation and expression of neurofilaments were promoted. More robustly, SBL showed a significant effect in potentiating the NGF-mediated neurite-inducing activity. A lot of neurological diseases are found to be associated with insufficiency of neurotropic factors, for example, depression [26] and Alzheimer's [27]. Neuronal cell death was found in depressed brain [28, 29]. Additionally, ROS also caused the hippocampal neurons loss [30]. In depressed brain, the secretion of neurotrophic factors could be reduced, and the neuron could not survive, grow, or differentiate normally [31]. For the property of potentiation effect on NGF-mediated neurite outgrowth, SBL would have the potential to be used to treat the differentiation obstruction caused by NGF deficiency. Besides, our preliminary results also suggested the induction of various trophic factors by SBL in cultured astrocytes (data not shown). 

Reports of antioxidant and neuroprotective effect of NRR have been published [32, 33]. Lyle et al. [34] reported that NRR alleviated the symptoms of chronic fatigue syndrome (CFS) with the role of antioxidant properties of NRR. Here, our results supported the notion that functional roles of SBL might be derived from its antioxidant properties. Phenolic components have strong antioxidant role as a hydrogen ion donor, and our colorimetric study showed that SBL extract contains relatively high amount of phenolic compounds. In addition, ferulic acid and linarin were shown to have protective effect on neurons [19, 21]. In our study, ferulic acid and linarin showed promising effect in reducing the intracellular ROS formation (data not shown). On the other hand, the volatile oils of SBL are a mixture of lipids, terpenoids, ketones, and phenols. *β*-Maaliene, aristolene, and calarene, isolated from NRR, were reported to have a strong sedative and sleep enhancing activity [35]: these chemicals account for 57.1% of total volatile oil of SBL. Moreover, a partially purified glycoside from NRR was able to induce the outgrowth of neurites and the expression of growth-associated protein 43 (GAP-43) [36]. Thus, the distinctive properties of SBL could depend on the synergistic effects of total phenols, terpenoids, and/or other chemical components in SBL.

## 5. Conclusion

In summary, our study focused on the neuro-beneficial role of SBL, a simple ancient TUM, in cultured PC 12 cells. We found that pretreatment of a chemically standardized SBL could protect the cells from oxidative stress, which could be mediated by scavenging of ROS formation and stimulating the Nrf2-ARE self-defense mechanism. In addition, SBL could increase the expression of neurofilaments in cultured PC 12 cells and potentiated the NGF-mediated neurite outgrowth. Lastly, we demonstrated that the inclusion of volatile oil is necessary for a complete function of SBL. Taken together, these results partially revealed the action mechanism of SBL by cell studies. 

## Supplementary Material

Supplementary Table 1: Calibration curve, LOD and LOQ of marker chemicals in NRR extract

## Figures and Tables

**Table 1:** Chemical composition of volatile oil from NRR.

Retention time (min)	Compound^a^	Molecular weight	RA (%)^b^
8.34	*β*-maaliene	204	7.9
8.62	9-aristolene	204	4.7
9.08	Calarene	204	37.9
9.29	*α*-maaliene	204	1.2
9.58	Guaia-6,9-diene	204	0.7
9.77	Valerena-4,1(11)-diene	204	6.6
10.79	*α*-humulene	204	1.6
12.49	Epi-*α*-selinene	204	1.5
14.64	*β*-lonone	192	2.1
15.08	4-epi-*α*-maaliol	222	1.9
19.24	Patchouli alcohol	222	5.5
19.68	Guaina-6,9-diene-4 *β* ol	220	3.7
22.44	Eudesma-3,11-dien-2-one	218	1.7
23.93	Aristolone	218	2.1

^a^The identified constituents are listed in their order of elution.

^b^RA indicates relative amount (peak area relative to the total peak area).

The extraction efficiency of NRR oil was over 95% within the 4 hours of distillation. In addition, the amount of extracted oil was 2.1 ± 0.28% (*n* = 4). The values are in mean of three individual experiments (*n* = 3). The SD values were less than 5% of the mean, not shown for clarity.
