# Supplementary material for: Song Bu Li Decoction, a Traditional Uyghur Medicine, Protects Cell Death by Regulation of Oxidative Stress and Differentiation in Cultured PC12 Cells
Source: Evid Based Complement Alternat Med. 2013 Sep 28;2013:687958. doi: 10.1155/2013/687958 (PMC3807552; doi:10.1155/2013/687958)
Supplement: Supplementary file 1 — Supplementary Table 1: Calibration curve, LOD and LOQ of marker chemicals in NRR extract [file 687958.f1.pdf]

**Supplementary Table 1. Calibration curve, LOD and LOQ of marker chemicals in NRR extract**

| Chemical | Calibration curve [a] | Correlation coefficient ($r^2$) | Linear range (μg/mL) | LOD [b] (μg/mL) | LOQ [c] (μg/mL) |
|---|---|---|---|---|---|
| **Ferulic acid** | y=55.833x+17.313 | 0.9996 | 0.09-50 | 0.10 | 0.19 |
| **Linarin** | y=25.093x+1.1019 | 1.0000 | 0.09-50 | 0.10 | 0.39 |

[a] Calibration curve was constructed by plotting the peak area versus the concentration of each analytes. The calibration curve was derived from six data points, *n* = 3, and the SD was < 5% of the mean.

[b] LOD refers to the limits of detection.

[c] LOQ refers to the limits of the quantification.
